# Evolving our support for early sharing

**DOI:** 10.1038/s41467-020-16788-3

**Published:** 2020-06-15

## Abstract

*Nature Communications* encouraged rapid dissemination of results with the launch of Under Consideration in 2017. Today we take one more step by offering an integrated preprint deposition service to our authors as part of the submission process.

Early sharing of results in preprints has been a familiar practice for some research communities for decades—physicists will know that the arXiv is turning 30 next year —and has grown in popularity with many other communities in the past 5–10 years, perhaps catalysed by the launch of bioRxiv, which now serves a large community of researchers in the life sciences.

While the word ‘preprint’ might sound archaic in a digitally-led world, the need for early sharing and discovery of results has never been greater, particularly at a time when a pandemic is sweeping the globe. We strongly believe in the importance of this practice to increase transparency in the research process and to speed up the dissemination and re-use of scientific results, while maintaining that a rigorous peer-review (normally coordinated as part of the publication process in a journal) is needed to validate the findings.

From today, our authors have the option to take advantage of In Review, a free preprint deposition service integrated with the submission process to our journal.

Nature Research, the portfolio of our sister journals, has long supported preprint deposition of the originally submitted version of manuscripts, and actively encourages it since last year. In order to further promote the practice of preprinting, *Nature Communications* launched an initiative in 2017 that allowed authors to list the preprints of papers hosted on any platform, and undergoing peer review at our journal, on our Under Consideration web page. The link from our page to the preprint is available for the length of the peer review process, and removed once a final decision is made.

We have been offering this option to our authors for two and a half years, and we are very happy to report that 59% of the authors who opted in ended up depositing a preprint of their work as a result of our initiative. The overall uptake has grown from 3% in 2017 to 7% in 2019, and has varied significantly by discipline, with 22% of participating papers in the physical sciences, and 78% in the life and biological sciences. Perhaps this uneven breakdown is a reflection of the more recent adoption of preprints by the latter community, when compared with the more mature practice in the physical sciences. Authors working in neuroscience have been by far the most receptive to this initiative, contributing 18% of all participating papers.

Wishing to facilitate greater adoption of preprints across the multidisciplinary scope of our journal, we are retiring Under Consideration to support a new early sharing tool with added functionalities for our authors.

From today, our authors have the option to take advantage of In Review, a free preprint deposition service integrated with the submission process to our journal. The preprint of the author’s original submission will be posted (with a permanent DOI, under a CC-BY licence) on the multidisciplinary platform hosted by our partner Research Square at the same time as the submission is being considered by our editorial team.

For authors who opt-in, the posting will happen when our editors decide whether to send the manuscript for external review. If the manuscript is sent to reviewers, the preprint on Research Square will be displayed as being ‘under review at Nature Research’ for as long as the manuscript is being considered. Once the manuscript is published, the preprint will link to the published version. When the preprint is posted, a HTML version of the manuscript will be created and displayed when original figures are available, making it more machine-readable—and hence easier to be discovered by search engines—than a PDF.

As part of the service offered by In Review, authors will be able to track the status of their manuscript thanks to a private peer review timeline, showing main events such as the recruitment of a reviewer or the receipt of a reviewer report. More information about In Review for Nature Research journals, including the minimum disclosures required, can be found here. At present, the In Review opt-in is available for submissions received via the *Nature Communications* portal, but not to authors transferring their paper from another Nature Research journal.

In Review has been offered on almost 300 Springer Nature journals since October 2018. This integrated preprint deposition service will hopefully make it easier, and thus more likely, for our authors to share their results early. Moreover, we believe that In Review will be particularly useful in serving communities that do not yet have an established discipline-specific preprint server, as well as providing more visibility for multidisciplinary works.

We continue to encourage preprint deposition on any community-recognised preprint server—it is solely the author’s choice to decide if and where they are going to post their results before the conclusion of the peer review process.

We are excited about this new step we are taking in support of early sharing. We will monitor reception of this new service by our authors, and continue to evolve our offering to them in response to the needs of the community.

